# The Role of Physicians in Digitalizing Health Care Provision: Web-Based Survey Study

**DOI:** 10.2196/31527

**Published:** 2021-11-11

**Authors:** Anja Burmann, Max Tischler, Mira Faßbach, Sophie Schneitler, Sven Meister

**Affiliations:** 1 Fraunhofer Institute for Software and Systems Engineering Dortmund Germany; 2 Witten/Herdecke University Witten Germany; 3 Hautärzte am Markt Dortmund Germany; 4 Bündnis Junge Ärzte Berlin Germany; 5 Helios Klinikum Duisburg Duisburg Germany; 6 Saarland University Hospital Homburg Germany; 7 German Society for Tropical Medicine, Travel Medicine and Global Health Hamburg Germany

**Keywords:** digitalization, digital transformation, health care, human factor, physicians, digital natives, web-based survey, digital health

## Abstract

**Background:**

Digitalization affects all areas of society, including the health care sector. However, the digitalization of health care provision is progressing slowly compared to other sectors. In the professional and political literature, physicians are partially portrayed as digitalization sceptics. Thus, the role of physicians in this process requires further investigation. The theory of “digital natives” suggests a lower hurdle for younger generations to engage with digital technologies.

**Objective:**

The objective of this study was to investigate the role of physicians in the process of digitalizing health care provision in Germany and to assess the age factor.

**Methods:**

We conducted a large-scale study to assess the role of this professional group in the progress of the digital transformation of the German health care sector. Therefore, in an anonymous online survey, we inquired about the current digital penetration of the personal working environment, expectations, attitude toward, and concerns regarding digitalization. Based on these data, we studied associations with the nominal variable age and variations across 2 age groups.

**Results:**

The 1274 participants included in the study generally showed a high affinity towards digitalization with a mean of 3.88 on a 5-point Likert scale; 723 respondents (56.75%) stated they personally use mobile apps in their everyday working life, with a weak tendency to be associated with the respondents’ age (η=0.26). Participants saw the most noticeable existing benefits through digitalization in data quality and readability (882/1274, 69.23%) and the least in patient engagement (213/1274, 16.72%). Medical practitioners preponderantly expect further improvements through increased digitalization across almost all queried areas but the most in access to medical knowledge (1136/1274, 89.17%), treatment of orphan diseases (1016/1274, 79.75%), and medical research (1023/1274, 80.30%).

**Conclusions:**

Respondents defined their role in the digitalization of health care provision as ambivalent: “scrutinizing” on the one hand but “active” and “open” on the other. A gap between willingness to participate and digital sovereignty was indicated. Thus, education on digitalization as a means to support health care provision should not only be included in the course of study but also in the continuing process of further and advanced training.

## Introduction

### Background

The theoretical description of digitalization in health care promises the potential to improve quality of care, save time, streamline documentation, and support access through natural forms of interface design [1,2]. Digitalization might thus enable the health care domain to cope with globally occurring challenges like cost, efficiency, complexity, and reform pressure [3]. However, according to an annual cross-sectoral investigation in Germany, the health care domain is not exploiting the potential to the same extent as are other domains [4]. Furthermore, within the domain (eg, between different hospitals), the digitalization status varies considerably [5,6]. Health care institutions, admittedly, contain particularities that distinguish them from classic value-creating companies: they heavily rely on “highly specialized human capital” [7]. Human acceptance factors in the health care context have been investigated using hospital information systems as an example [8]. The heterogenous digitalization success of health care institutions calls for further investigation into the underlying causes and effects of this variability [9]. The German health care system is decisively governed in a self-administered manner [10]. Although all self-governing institutions are under the legal supervision of the state and are bound by the state's framework legislation, they are not under the professional supervision of the state. Representatives of health insurance companies, health care service providers, and patient representatives negotiate and determine medical services that are covered by the statutory health insurance. Advocacy groups thus have an important role in balancing the stakeholder’s interests for the benefit of the common good. Additionally, several studies have pointed out the importance of humans as potentially the greatest obstacle to or the greatest promoter of digitalization in health care processes [11-13]. In the professional and political literature, physicians are partially portrayed as digitalization skeptics [14]. Individual physicians’ organizations generally position themselves against efforts to increase the digitalization of health care processes [15,16], while other studies present a low digital penetration rate and a need for action [17,18]. Thus, the aim of this study was to further investigate the role of physicians in the process of digitalization as one of the key stakeholder groups in health care provision.

### Prior Work

Digitalization is a disruptive change that affects all areas of society [19]. However, there is currently no consensus on a generally applicable definition of this term. With regard to an original technical understanding, digitization means the “conversion of analogue data (image, text, sound, etc) into digital data” [20]. Definitions of digitalization range from the “replacement of analogue service provision […] in whole or partly by service provision in a digital, computer-manageable” way [21], to the integration of all involved actors and data through digital technologies that influences the entire value chain [22]. Regarding health care organizations, Meister et al [19] describe digitalization as a “continuous change process,” which combines the incorporation of digital technology and the ability to constantly adapt to changing conditions.

The importance of the human factor in health care processes is highlighted in the concept of health care–providing institutions as “expert organizations” [11,13,23]. Expert organizations are defined as “knowledge and competence-intensive service organizations whose value creation is primarily based on the recruitment, refinement and use of highly specialized human capital” [7]. Experts are thus individuals who are highly qualified, have a strong position in their institution, and strongly identify with their profession. Furthermore, they have a high degree of autonomy in decision-making and create complex services or products [23]. The integration of interprofessional knowledge and skills of experts participating in the clinical treatment process constitutes “the most important capital” in health care provision [23]. Digital process support requires a full integration across all contributors and change on different levels of hitherto established structures of health care institutions [24]. Child [25] states that experts are especially likely to be suspicious toward change of their established routines. As stated above, individual physicians’ organizations, as stakeholders of self-administration, have raised concerns regarding digitalization [15,16]. This might partly be due to a general skepticism toward change in humans [26]. In the field of digitalization, however, the term “digital natives” is often used, which assumes a lower hurdle for younger generations to engage with digital technologies [27]. The role that the factor of age has indeed been investigated in the field of technology acceptance in general [28] but also with regard to the digitalization of hospitals. Hospital employees themselves suspect age to be a decisive factor in whether the digital transformation of their working environment is accepted or not [29].

### Objective of This Study

The purpose of this study was to examine the role of physicians in the digital transformation of health care with a specific focus on the variable of age. Therefore, the following 3 research questions were investigated: (1) How do physicians perceive opportunities and risks of the digital transformation of their working environment? (2) How do physicians see their own role in digitalizing health care provision? (3) What role does age play in the perception of digitalization of health care provision and the personal role within this process?

In order to examine these issues, a nationwide survey among physicians in Germany was conducted.

## Methods

### Survey Design

The survey was designed in an iterative manner by scientists in the field of digital health and members of the Bündnis Junge Ärzte (BJÄ, Alliance of Young Physicians), a union of representatives of young physicians from 25 medical associations and medical societies in Germany. We followed the survey principles outlined by Dillman et al [30] and Schleyer and Forrest [31], while the results are reported in accordance with Eysenbach [32]. The survey design resulted in a structured format comprising a maximum of 42 questions, with adaptive questioning being used to reduce complexity and volume for the participants. Single- and multiple-choice questions were included with answer types assigned to nominal, ordinal, and ratio scales. Free-text fields were provided for further explanatory comments. The full translated questionnaire can be found in the Multimedia Appendix 1.

On the survey landing page, we describe the survey topics and length, goals and target group, and the inquiring organizations, and provide information on the data handling according to the European General Data Protection Regulation (GDPR). The survey was voluntary, nonincentivized, and fully anonymous. None of the participant information requested could be used to identify the participant, and no technical identifiers (eg, IP address) were stored. To start the survey, participants were required to express consent to the procedure. The first survey section included demographic questions related to the respondent’s age, gender, professional position and type of employment, medical specialization, and general digital affinity derived from items provided by the technology affinity questionnaire from Karrer et al [33]. The following section “Status Quo,” comprised questions regarding degree of digital process support in the respondent’s current working environment, including internal and intersectoral data handling. The medical process steps queried in this section were derived from the best practice report provided by Kılıç [34], which describes digital health care processes, as well as the approach by Burmann et al [35], who describe different maturity states of digital health care provision. Moreover, the already noticeable benefits through digitalization and the areas of untapped potential were addressed. These areas, where advantages through digitalization are anticipated, were adapted from the industry and hospital 4.0 paradigm [36,37]. Following this, the role of medical professionals was examined. In response to the controversial description of medical practitioners as, by profession, not being capable of orchestrating digitally supported health care supply chains [38], the view on hindrances to digitalization of the mentioned group was queried. Additionally, the respondents were asked to assess their own familiarity with current technological, processual, and legal topics with regard to digitalization of the German health care sector. The following section, “Mobile Health Apps,” was dedicated to general professional mobile app use and digital health apps. The latter was involved due to its facilitation of medical prescriptions for digital health apps (Digitale Gesundheitsanwendungen [DiGA]) by law through the digital health care act (Digitale-Versorgung-Gesetz [DGV]), which came into effect in Germany just when the survey was launched [39]. The last survey section, “Future,” detailed the respondents’ perspectives on the future of digital health supply, including expectations and the personal role the professionals within this current change. Each survey section was presented on a single page, resulting in a total number of 5 pages including the welcome message. A total of 42 questions were partitioned across 4 questionnaire pages. Comprehensibility, usability, and technical functionality were tested before the survey launch with a group of members of the BJÄ.

### Recruitment

The target group of the survey was practicing and prospective physicians. In order to effectively use the distribution channels of the BJÄ for acquiring a convenience sample, the survey was held open. To prevent multiple participation, a cookie was set with submission of the questionnaire. The survey was administered from October 16, 2020, to December 18, 2020, via the online survey platform LimeSurvey. During this period, the survey was publicly available and repeatedly announced through various online and personal channels, including social media accounts (Twitter, LinkedIn, Facebook), press releases, and mailing lists from the BJÄ and its 25 member associations, as well as magazines and newsletters for the health care sector. Furthermore, the personal approaches of the professional networks of the actors involved were used. We provided a dedicated URL redirect which led to the survey via a link. The first contact points with participants were professional networks, or a personal or direct approach via medical associations, all mainly through online channels.

### Data Exclusion

We included only those questionnaires that were complete and from respondents with a professional background as medical practitioners. The latter included physicians either in training or in practice in the health care sector. Responses from medical practitioners in retirement or employed in the industrial context, as well as actors with other professional backgrounds were thus excluded from further analysis. From 1940 initial questionnaires, 651 were excluded due to incompletion or missing values, resulting in a completion rate of 66.44%. A further 15 questionnaires were then excluded due to the aforementioned exclusion criteria, resulting in 1274 included data sets.

### Data Analysis

The main outcome variables of the survey were the perceived digitalization hindrances, the anticipated role of digitalization in the future health care process, and the respondent’s role within this change process. The first aspect was assessed through multiple-choice questions, while the latter 2 were assessed with both single-choice and multiple-choice questions. All mentioned outcome variables were assigned to nominal scales. Descriptive variables included nominal scales (gender, working environment, medical specialization), ordinal scales (professional level, Likert-type digital affinity), interval (Likert scale digital affinity), and ratio scales (age). In order to examine the relation between the outcome variables and age as the primary covariate, we carried out statistical parametric tests for metric scales and nonparametric tests for investigating the association of categorical and metric variables [40]. Depending on the respective scale of the covariate, Pearson correlation coefficient [41], *t* test [42], effect analysis with Cohen *d* [43], and η coefficient [44] was calculated. For accompanying questions, the percentage of respondents who chose each item was calculated. The descriptive data analysis was carried out using Microsoft Excel (Microsoft Corp). For the investigation of associations via Pearson correlation coefficient and *t* test, along with effect analysis with Cohen *d* and η, the open source software PSPP (GNU project) was used. For parametric testing via Pearson correlation coefficient, the assumption of linearity, related pairs, absence of outliers, and suitable measurement scales were investigated. For the application of *t* tests, the assumptions of suitable measurement scales, adequacy of sample size, and homogeneity of variance were examined [45].

## Results

### User Statistics

The total of 1274 complete and included responses comprised 567 (44.51%) female respondents. The age of the respondents ranged from 22 to 67 years (mean 45.09, SD 12.06). The professional level of the respondents included first level, medical students (24/1274, 1.88%); second level, physicians in specialist training (328/1274, 25.75%); third level, medical specialists with <5 years professional experience (180/1274, 14.13%); fourth level, medical specialists with >5 years professional experience (732/1274, 57.46%); and other (10/1274, 0.78%). The 2 major shares of respondent’s working environment was split approximately evenly, with one-half working in clinical environments (593/1274, 46.55%) and one-half in physician’s offices (594/1274, 46.62%), with 87 others (6.83%). The 5 most-represented medical specializations were general internal medicine (184/1274, 14.44%), dermatology and venerology (138/1274, 10.83%), ophthalmology (133/1274, 10.44%), urology (122/1274, 9.58%), and general medicine (103/1274, 8.08%). Further demographic data of the respondents are depicted in Table 1.

**Table 1:** Respondent’s demographics (N=1274).

Characteristic				Value, n (%)
**Gender**				
	Female			567 (44.51)
	Male			706 (55.42)
	Other			1 (0.08)
**Age**				
	≤35 years			382 (29.98)
	36-45 years			290 (22.76)
	46-55 years			276 (21.66)
	≥56 years			326 (25.59)
**Professional level**				
	First: medical student			24 (1.88)
	Second: specialist training			328 (25.75)
	Third: specialist <5 years			180 (14.13)
	Fourth: specialist >5 years			732 (57.46)
	Other			10 (0.78)
**Working environment**				
	**Clinic**			593 (46.62)
		University hospital	192 (32.43)	
		Public hospital	165 (27.87)	
		Nonprofit hospital	134 (22.64)	
		Privat hospital	92 (15.54)	
		No answer	9 (1.52)	
	**Physician’s office**			594 (46.55)
		Self-employed	465 (78.28)	
		Employee	129 (21.72)	
	Other			87 (6.83)
**Volume of employment**				
	Full-time			1008 (79.12)
	Part-time			227 (17.82)
	Marginal employment			13 (1.02)
	No answer			26 (2.04)

The demographic factor “digitalization affinity” was also measured. For this, 4 suitable theses from a general technology affinity questionnaire [33] were taken and adapted to the focus of digitalization. These 4 theses included affinity toward exploring digital services, perceived ease of access, impact on everyday convenience, and impact on communication. All these were queried in a 5-point Likert-type scale (“strongly disagree”=1 to “totally agree”=5). After investigating internal consistency (Cronbach α=.68) [46], we combined these items into a single Likert scale by calculating the mean value per respondent [47].

For further investigation, we also split the respondents into 2 groups: based on the age limit of the BJÄ and the definition of the German medical associations, we placed participants who were 45 years of age and younger into group 1 (672/1274, 52.75%) and those who were older than 45 years into group 2 (602/1274, 47.25%).

### Descriptive Outcomes

The digital affinity variable, comprising 4 five-point Likert-type items within a Likert scale resulted in a moderate tendency toward a positive perception of digitalization. The mean score across all respondents was 3.88 (SD 0.67). In the following subsections (*Status Quo*, *Mobile Health Apps*, and *Future*), we present a descriptive analysis of these 3 areas of the questionnaire.

#### Status Quo

First, we identified the status quo of use of digital systems in the respondents’ everyday working life. We queried the 4 segments of internal process support (including applications and data administration), interorganizational data exchange, professional communication, other digital services for internal organization (that do not directly concern patients, such as professional training or e-learning, duty planning, worktime recording), and other services addressed to patients (eg, appointment scheduling, virtual consultation hours, medication plan, access to patient data, mobile apps).

**Figure 1:**
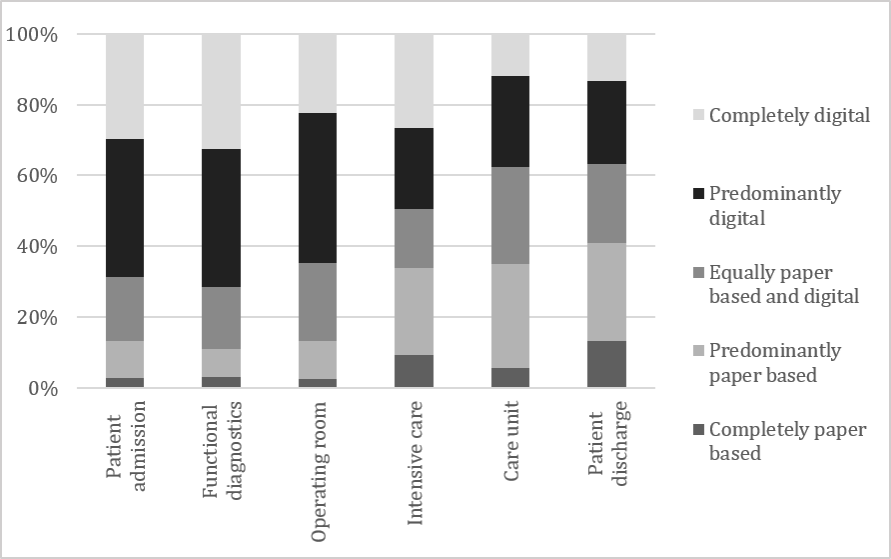
Status quo: respondents' assessment of digitalization of internal processes.

The digitalization of internal processes was led by functional diagnostics (radiography, laboratory), with 749/1187 (68.72%, adjusted by the share of respondents who stated that this was not relevant for them) respondents indicating that they organize completely or predominantly digitally. This was directly followed by the areas of patient admission (749/1090, 68.71%), operating room (398/616, 64.61%), and intensive care (207/418, 49.52%). Care unit (240/637, 37.68%) and patient discharge (264/716, 36.87%) were the least digitally organized areas. The adjusted percentages of responses are shown in Figure 1.

Interorganizational data exchange still is a primarily paper-based process, as only 132 of 1259 respondents (10.48%) stated they receive data completely or predominantly digitally from other service providers, while 161 of 1258 (12.80%) transfer data themselves mainly in a digital format to other service providers.

As expected, regarding professional communication, the phone call was still the predominant tool for interaction, as 1248 of 1274 respondents indicated using it for professional communication (97.96%). Fax (1082/1274, 84.93%), mail (967/1274, 75.90%), and email (976/1274, 76.61%) were also used by a substantial majority. Meanwhile, medical platforms (115/1274, 9.03%), messaging apps for specific medical purposes (142/1274, 11.15%), and generic messaging apps (332/1274, 26.06%) were ranked at the bottom of the list.

Patient distant digital services use was relatively widespread: 1006 participants (78.96%) stated that they used digital services for professional training or e-learning, 776 (60.91%) planned their duty in a digital system, and 579 (45.45%) recorded their worktime electronically.

Interestingly, services addressed to patients did not show a high degree of dissemination. Digitalized appointment scheduling ranked highest, with 24.49% (312/1274) of the respondents stating that they offered this service, followed by the provision of an electronic medication plan (235/1274, 18.45%), access to personal data (228/1274, 17.90%), virtual consultation (203/1274, 15.93%), and mobile apps (47/1274, 3.67%). The provision of none of these services without mentioning alternatives in use was the only option selected more frequently (580/1274, 45.53%). One question was then aimed at the proactive offering of health-related data for assessment through patients themselves, acquired by, for instance, wearables or apps. Of the 1274 respondents, 51 (4%) indicated experiencing this regularly, 197 (15.46%) occasionally, 448 (35.16%) sporadically, and 553 (43.41%) had never encountered this situation. Of the 696 respondents who had encountered self-acquired patient data to a varying extent, 41 (5.90%) generally refused to incorporate this kind of information into their medical investigation, 314 (45.11%) stated they verify only acutely relevant data, and 341 (48.99%) indicated being generally open to data from consumer products provided by the patient.

**Figure 2:**
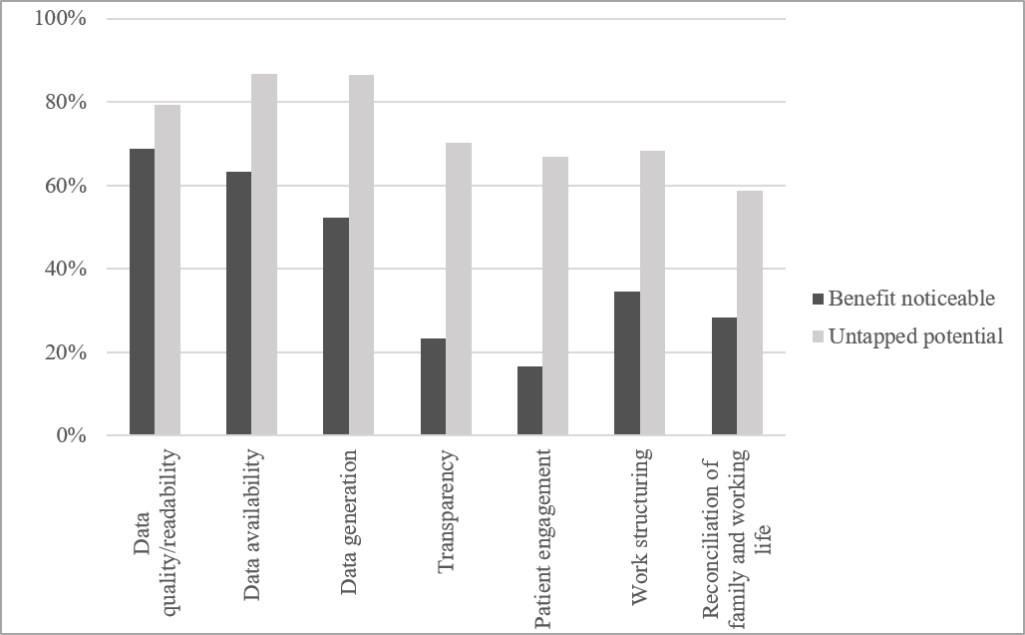
Shares of respondents who see noticeable benefits and untapped potential through digitalization across different categories.

We queried perceptions regarding the already existing benefits of digitalization and untapped potential in the 7 categories of data quality and readability, data availability, data generation, transparency, patient engagement, work structuring, and reconciliation of family and working life, and responses varied considerably. Affirming the comparatively low usage of digital services for patients, only 213 of 1274 respondents (16.72%) already noticed benefits through digitalization in the category of patient engagement. An only slightly higher perception of utility was indicated for transparency, while data quality and readability and data availability were indicated to have received the most benefit thus far. However, the believed untapped potential exceeded the already noticeable benefit in all queried categories. Data availability, generation, and quality or readability ranked highest while optimism for digitalization improving everyday working life (working structure and reconciliation of family and working life) was also present, but not quite on the same level. The detailed data concerning the perceived benefits and potential of digitalization are displayed in Figure 2.

The next 2 multiple choice questions queried obstructions and constraints in digitalization regarding the user and technology side. For the user side, we asked the respondents to indicate whether they perceived 5 different items to be a “major hindrance” for digitalization. Almost half of the respondents (626/1274, 49.14%) stated a lack of noticeable saving of time to be a major impeding factor. Slightly fewer respondents considered insufficient digital literacy or sovereignty (530/1274 41.60%), fear of surveillance (508/1274, 39.87%), and an unwillingness to change (461/1274, 36.19%) to also be hindrances. Fear of loss of importance was indicated to be a major limiting factor by 99 respondents (7.77%), and 210 respondents stated they did not perceive these hindrances to be present in themselves or their age group. When “other” was indicated, these responses referred to data security concerns, loss of trust between patients and physicians, and insufficient user integration.

The most-frequently chosen major technical hindrance was “insufficient system integration” (798/1274, 62.64%). Almost half of the respondents perceived insufficient software functionality (575/1274, 45.13%) to be a major issue, followed by insufficient hardware (503/1274, 39.48%), insufficient budget (453/1274, 35.56%), legal concerns regarding the exchange of medically sensitive data (341/1274, 26.77%), and insufficient cooperation by system providers (247/1274, 19.39%). When “other” was indicated, these response referred to data security concerns, system availability or performance issues, and a lack of user-centered system design.

**Figure 3:**
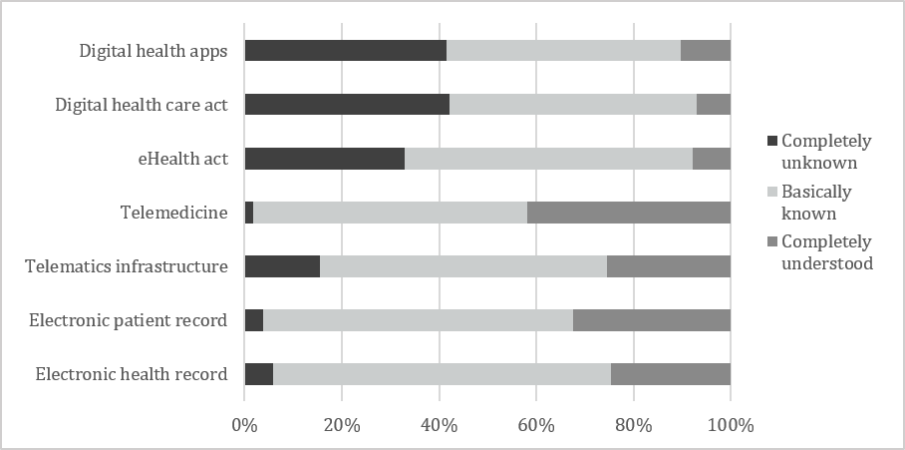
Respondents' assessment of familiarity with current trending digitalization topics.

Subsequently, the respondents provided an assessment of their familiarity with the current trending topics regarding digitalization of the health care sector in Germany. This included electronic health and electronic patient records, telematics infrastructure, telemedicine, the eHealth act, the digital health care act (DGV), and digital health apps (DiGA). For each topic, participants were required to indicate if it was completely unknown, basically known, or completely understood by them. The distributions of responses across these 7 topics are summarized in Figure 3. It is important to note that at the time of the survey (November 2020 to December 2020) some currently trending topics (eg, digital health apps, the digital health care act, and eHealth act) were less well known than were others.

#### Mobile Health Apps

Of the 1274 respondents, 723 (56.75%) stated that they personally used mobile apps in their everyday work life. The most mentioned field of use was pharmaceutical information (516/723, 71.37%) and diagnosis (386/723, 53.39%), followed by training (317/723, 43.85%) and communication (300/723, 41.49%). When asked whether they trust in digital health apps, 425 stated yes (33.36%), 196 stated no (15.38%), and 653 stated “that depends” (51.26%).

Only a small portion of respondents (223/1274, 17.50%) stated that they had recommended specific mobile health apps to their patients. Of the 1051 respondents who had not yet recommended an app, 420 (39.96%) stated that their reasons for not having done so included “insufficient validity”, 286 (27.21%) stated it was “not relevant in my area,” and 266 (25.31%) indicated “insufficient data protection.” However, 80.46% (1025/1274) of the participants expect mobile or digital health apps to play a role in health care provision in the future.

Regarding the main sources of information for mobile or digital health apps, 812 (63.74%) indicated medical societies as the main source, 671 (52.67%) the internet, and 622 (48.82%) colleagues. A much smaller proportion of respondents indicated public bodies (182/1274, 14.29%) and developers (104/1274, 8.16%) as playing important roles in information acquisition.

#### Future

In the section, “Future,” the survey participants were first asked to rate their expectation for the impact of digitalization on 10 health care provision–related areas from “worsening” over “no change” to “improving.”. The highest positive expectations were shown in the areas “access to knowledge” (1136/1274, 89.17%), “medical research” (1023/1274, 80.30%), and “treatment of rare diseases” (1016/1274, 79.75%). The greatest doubts were expressed in the areas of “physician-patient relationship” (397/1274, 31.16%), “administration” (265/1274, 20.80%), and “attractiveness of the profession” (237/1274, 18.60%; see Table 2).

**Table 2:** Expected impact of digitalization on health care provision (N=1274).

Area	Worsening, n (%)	No change, n (%)	Improving, n (%)
Early detection of diseases	51 (4)	549 (43.09)	674 (52.91)
Medical quality	156 (12.24)	422 (33.12)	696 (54.63)
Access to knowledge	10 (0.78)	128 (10.05)	1136 (89.17)
Treatment of rare diseases	8 (0.63)	250 (19.62)	1016 (79.75)
Administration	265 (20.80)	207 (16.25)	802 (62.95)
Patient adherence	91 (7.14)	691 (54.24)	492 (38.62)
Physician-patient relationship	397 (31.16)	672 (52.75)	205 (16.09)
Interdisciplinary collaboration	78 (6.12)	334 (26.22)	862 (67.66)
Attractiveness of the profession	237 (18.60)	644 (50.55)	393 (30.85)
Medical research	20 (1.57)	231 (18.13)	1023 (80.30)

Subsequently, the respondents rated their attitude towards upcoming changes through digitalization from “mainly positive” (567/1274, 44.51%) to “with mixed feelings” (557/1274, 43.72%) to “mainly negative” (130/1274, 10.20%).

When asked for multiple adjectives to describe their personal role in digitalizing health care provision, 36.50% (465/1274) of respondents assessed themselves as “scrutinizing,” 30.06% (383/1274) as “active,” 29.51% (376/1274) as “open,” and 25.20% (321/1274) as “critical.” Only 1.73% (22/1274) stated that they were “indifferent.”

### Investigation of Age Associations

A Pearson correlation coefficient (*r*=–0.30; *P*<.001) revealed a significant negative linear relationship between age and the digital affinity variable. The Likert scale resulted in a mean of 4.06 for group 1 (SD 0.55) and 3.68 for group 2 (SD 0.72). A significant difference between the 2 groups was found in a 2-tailed *t* test (t_1122_=10.64; *P*<.001) with a medium effect size (Cohen *d*=0.61). Inhomogeneity of variances was presumed based on a Levene test (*P*<.001).

#### Status Quo

We assumed that the already existing penetration of digital systems, mainly queried in the section, “Status Quo,” was substantially dependent on other factors (eg, working environment, financial situation of the employing organization, career stage). Thus, we focused this investigation on areas presumably in the sphere of influence of the respondents.

Regarding the communication medium of choice, no noticeable differences were found between the 2 age groups. Fax was used by 86.61% (582/672) of group 1 and 83.01% (500/602) of group 2, specific medical messaging apps were used by 10.42% (70/672) of group 1 and 11.96% (72/602) of group 2, and generic messengers were used by 26.34% (177/672) of group 1 and 25.75% (155/602) of group 2.

The perception of already noticeable benefit through digitalization was almost equally distributed in the 2 age groups. In the 7 queried categories (data quality and readability, data availability, data generation, transparency, patient participation, work structuring, and reconciliation of family and working life) an average of 41.48% (279/672, SD 22.71%) of age group 1 stated that they already noticed benefits of digitalization while an average of 41.03% (247/602, SD 18.53%) in age group 2 stated the same. Moreover, the *η* coefficient showed no or negligible association between age and the assessments of noticeable benefits within these 7 categories. However, when asked about the untapped potential of digitalization, the 2 age groups showed differences. In age group 1, an average of 83.25% (559/672, SD 8.71%) saw untapped potential across these categories, while the average in age group 2 was 64.29% (387/602, SD 13.06%). A 2-tailed *t* test (t_12_=3.20; *P*=.003) underlined the significance of this difference between the 2 groups, and Cohen *d* showed a strong effect size (*d*=1.71). Homogeneity of variances was asserted using a Levene test, which showed that equal variances could be assumed (*P*=.17). Additionally, the singular assessment of each category, except for “data generation,” showed an association with age. The *η* associations of noticed benefits, perceived untapped potential, and the age of the respondents are depicted in Table 3, along with the number of affirmations per group.

**Table 3:** Association between noticed benefits and potentials across categories and affirmation numbers per group.

Category		Association with age (*η*)	Group 1, n (%) (N=672)	Group 2, n (%) (N=602)
**Noticeable benefits**				
	Data quality/readability	0.08	486 (72.32)	396 (65.78)
	Data availability	0.05	449 (66.82)	362 (60.13)
	Data generation	0.08	372 (55.36)	298 (49.50)
	Transparency	0.00	160 (23.81)	139 (23.09)
	Patient participation	0.03	115 (17.11)	98 (16.28)
	Work structuring	0.11	201 (29.91)	241 (40.03)
	Reconciliation of family and working life	0.09	168 (25)	195 (32.39)
**Untapped potential**				
	Data quality/readability	*0.26* ^a^	601 (89.43)	417 (69.27)
	Data availability	*0.27* ^a^	638 (94.94)	474 (78.74)
	Data generation	0.16	614 (91.37)	494 (82.06)
	Transparency	*0.23* ^a^	533 (79.32)	367 (60.96)
	Patient participation	*0.25* ^a^	518 (77.08)	339 (56.31)
	Work structuring	*0.25* ^a^	534 (79.46)	343 (56.98)
	Reconciliation of family and working life	*0.27* ^a^	478 (71.13)	275 (45.68)

^a^Italics indicate a significant difference between the 2 age groups.

The attitude toward patient-provided consumer data was generally positive in both groups: of the respondents who had encountered this, 94.38% (336/356) in group 1 were willing to incorporate these data into their medical investigation while 93.82% of group 2 (319/340) showed the same willingness. A negligible association between age and willingness was found to be related to the willingness to incorporate this type of data (*η*=0.13).

Regarding the perception of major hindrances for digitalization on the user side, the 2 groups showed differences in 3 categories. The ratings in lacking noticeable saving of time, insufficient digital literacy or sovereignty, and no perception of such hindrances in themselves and their age group showed a weak association in the *η* coefficient with the nominal variable of age (see Table 4).

**Table 4:** Association of respondents’ perception of major hindrances for digitalization with age and the agreement numbers per age group.

Hindrance		Association with age (*η*)	Group 1, n (%) (N=672)	Group 2, n (%) (N=602)
**User side**				
	Insufficient digital literacy/sovereignty	*0.21* ^a^	223 (33.18)	307 (51)
	Lack of willingness to change	0.01	247 (36.76)	214 (35.55)
	Lack of noticeable saving of time	*0.24* ^a^	260 (38.69)	366 (60.80)
	Fear of loss of importance	0.02	57 (8.48)	42 (6.98)
	Fear of surveillance	0.15	222 (33.04)	286 (47.51)
	No such hindrances	*0.27* ^a^	172 (25.60)	38 (6.31)
**Technology side**				
	Insufficient hardware	*0.26* ^a^	343 (51.04)	160 (26.58)
	Insufficient software functionality	0.08	323 (48.07)	252 (41.86)
	Insufficient system integration	0.10	444 (66.01)	354 (58.80)
	Insufficient budget	0.03	186 (27.68)	144 (23.92)
	Insecurity with legal framework regarding data exchange	0.05	164 (24.40)	177 (29.40)
	Insufficient cooperation by system providers	0.12	99 (14.73)	148 (24.58)
	No such hindrances	0.08	8 (1.19)	16 (2.66)

^a^Italics indicate a significant difference between the 2 age groups.

**Figure 4:**
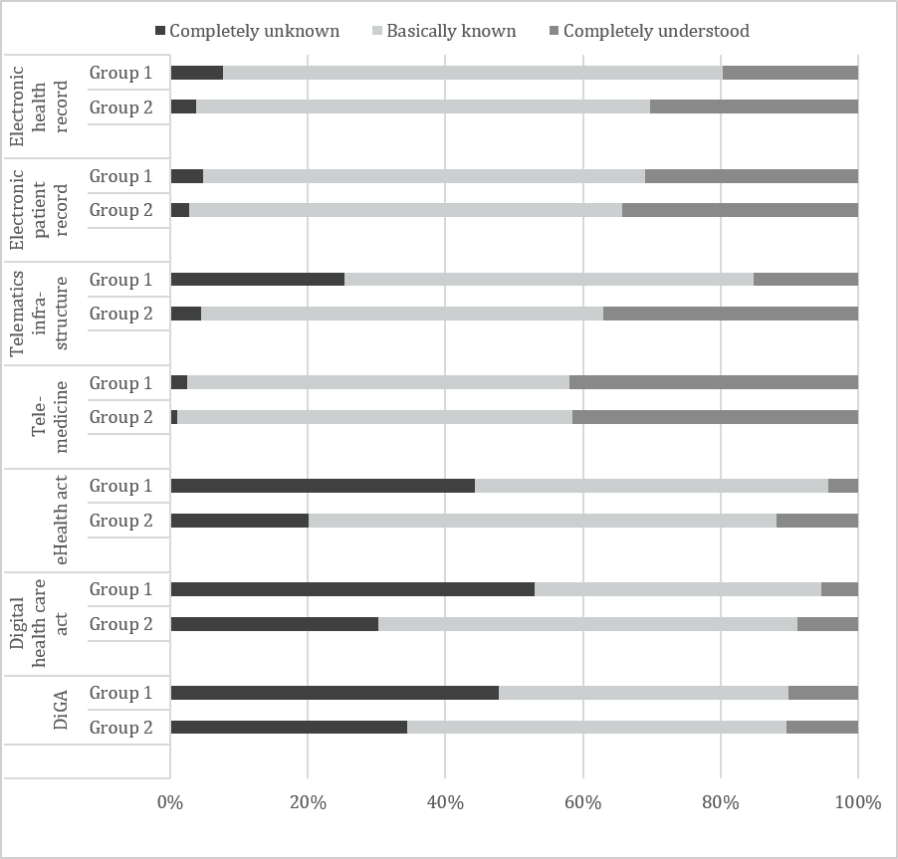
Respondents' assessment of familiarity with current trending digitalization topics by age group. DiGA: Digitale Gesundheitsanwendungen.

The distribution of familiarity with current trending topics regarding digitalization of the health care sector in Germany across age groups 1 and 2 is displayed in Figure 4. Age group 1 consider themselves to be less well informed across all topics, except telemedicine. However, the association of “informedness” with age was negligible in all categories except telematics infrastructure (*η*=0.36), the eHealth act (*η*=0.31), and the digital health care act (*η*=0.25), where a weak association between age and familiarity was found.

#### Mobile Health Apps

Respondents’ personal use of mobile apps in their everyday working life was more common in age group 1 (456/672, 67.86%) than in group 2 (267/602, 44.35%), which showed a weak tendency (*η*=0.26) in the association with the respondents’ age.

The fields of use also showed differences between the 2 groups. The use of mobile apps for information on pharmaceuticals and as a diagnosis aid showed a weak association with the age (*η*=0.29 and *η* =0.23, respectively; Table 5.)

**Table 5:** Association of professional usage fields of mobile apps and occurrence per age group.

Professional usage fields of mobile apps	Association with age (*η*)	Group 1, n (%) (N=672)	Group 2, n (%) (N=602)
Communication	0.03	148 (41.49)	152 (56.93)
Training	0.13	195 (42.76)	122 (45.69)
Information on pharmaceuticals	*0.29* ^a^	354 (77.63)	162 (60.67)
Diagnosis	*0.23* ^a^	264 (57.89)	122 (45.69)

^a^Italics indicate a significant difference between the 2 age groups.

Regarding trust in digital health apps, we also saw a tendency of group 1 to have more confidence in these (yes: 305/672, 45.39%; no: 42, 6.25%; that depends: 325, 48,36%) compared to group 2 (yes: 120/602, 19.93%; no: 154, 25.58%; that depends: 328, 54.49%). The *η* coefficient (*η*=0.35) showed a weak association with the age.

The recommendation rate of mobile health apps to patients did not relate noticeably with respondent age (*η*=0.02). As a reason for not having recommended an app, group 1 mentioned “insufficient data protection” (84/672, 15.19%) at a lower proportion than did group 2 (182/602, 36.55%) with *η*=0.21 for the nominal association with age, while other reasons showed no or negligible association with the age. Moreover, the belief that mobile apps will be relevant for future health care provision was somewhat stronger in group 1 (603/672, 89.73%) than in group 2 (422/602, 70.10%), with a weak relation with the nominal variable (*η*=0.21).

As an important source of information, the internet (*η*=0.14), public bodies (*η*=0.09), medical societies (*η*=0.15), and developers (*η*=0.04) were valued without considerable associations with the age. Only the selection of colleagues as an important information source had a slightly increased importance in group 1 (398/672, 59.23%) compared to group 2 (224/602, 37.21%; *η*=0.26).

#### Future

**Figure 5:**
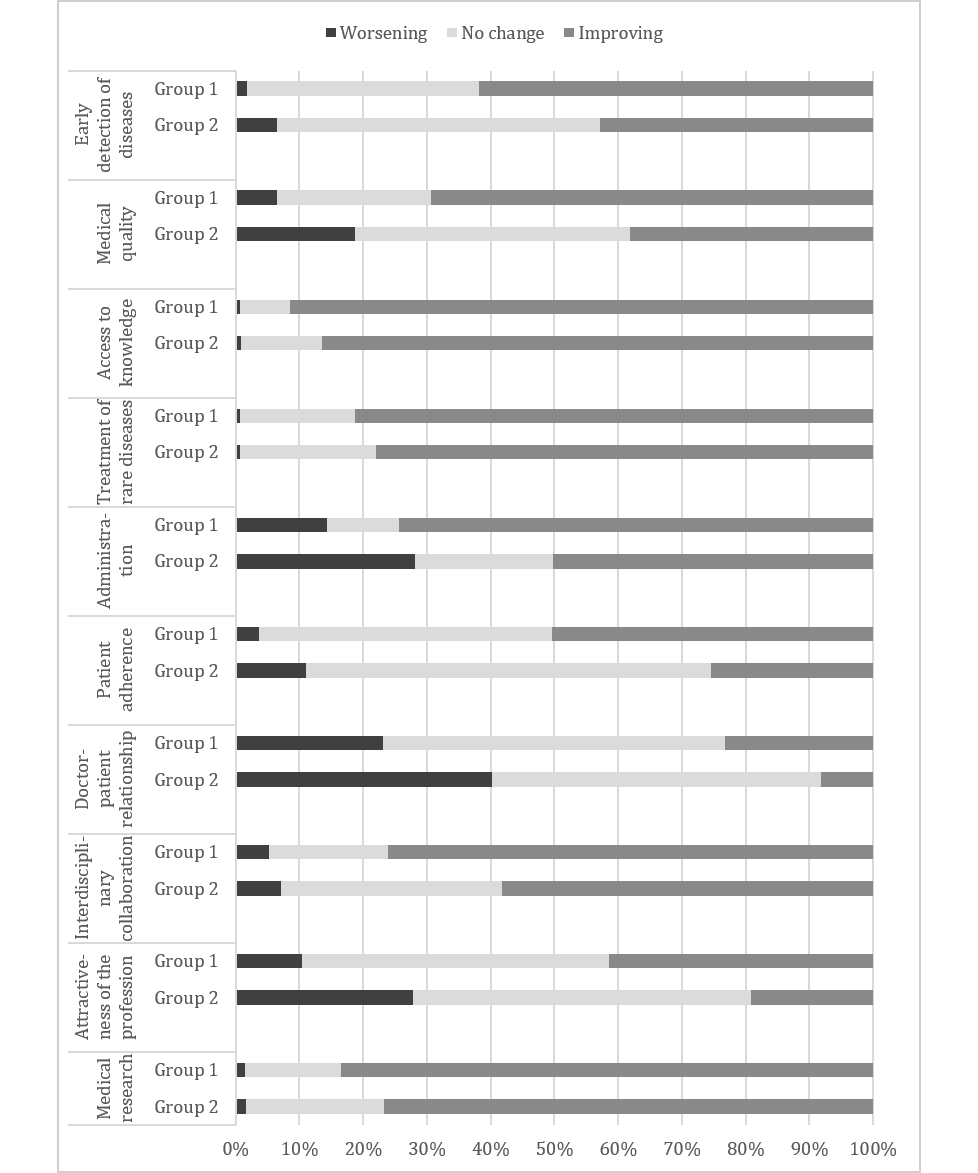
Expected impact of digitalization on health care provision by age group.

The assessment of the expected impact of digitalization on 10 health care provision–related areas showed a generally positive attitude. Mixed emotions became apparent regarding the physician-patient relationship, administration, and the attractiveness of the profession. In Figure 5, the assessments are displayed by age group. The areas “access to knowledge,” “treatment of rare diseases,” and “medical research” were assessed equally by both groups. However, weak associations between assessment and age were found in “medical quality” (*η*=0.30), “attractiveness of the profession” (*η*=0.28), “administration” (*η*=0.27), “patient adherence” (*η*=0.27), “physician-patient relationship” (*η*=0.25), “early detection of diseases” (*η*=0.21), and “interdisciplinary collaboration” (*η*=0.21).

The subsequent rating of the personal attitude towards upcoming changes through digitalization also showed a weak association with the age (*η*=0.36), with a rather positive trend in age group 1 (mainly positive: 399/672, 59.38%; with mixed feelings: 241/672, 35.86%; mainly negative: 25/672, 3.72%) compared to age group 2 (168/602, 27.92%; 316/602, 52.49%; and 105/602, 17.44%, respectively). For adjectives used to describe self-perceived roles, only the description of “critical” (group 1: 91/672, 13.54%; group 2: 230/602, 38.21%) and “open” (group 1: 260/672, 28.69%; group 2: 116/602, 19.27%) showed an association, albeit a weak one, with age (*η*=0.27 and *η*=0.21, respectively).

## Discussion

### Principal Results

In this study, one of the main stakeholder groups when it comes to digitalizing health care provision, physicians, showed a general affinity toward digitalization, with a negative linear tendency with decreasing age of the participants. Considering length and complexity of the questionnaire, the completion rate of 66.44% confirms a high interest of the sample in the enquired topic [48]. Survey dropout mainly occurred on survey pages 0 (welcome and consent, 223/666, 33.48%) and 1 (352/666, 52.85%).

The penetration of already existing digital process support was found to be heterogeneous for intraorganizational process areas, while interorganizational processes in general are still primarily paper based.

Also, digital services for professional communication have not yet reached a high adoption rate, with no association to users’ age. Other services for organizing working life, such as duty planning or e-learning, show relatively widespread use. This contrasts with digital offers for patients, which reach a maximum usage rate of a quarter of the respondents for appointment scheduling, while other services show much lower proportions. Meanwhile, more than half of the respondents indicated that they had experienced proactive offering of self-acquired data by patients, with the majority being willing to incorporate these data into medical decision-making. The age groups did not show differences in this regard.

The greatest perceived benefits of digitalization were data quality and readability. Perceived benefits were not associated with respondents’ age. However, participants saw untapped potential in all queried areas, with a relation with age to all categories except for data generation.

As major hindrances for digitalization, participants indicated a lack of a noticeable saving of time, followed by insufficient digital literacy or sovereignty as the dominant human factors. The association with the nominal variable of age in the category of insufficient digital literacy or sovereignty and no perception of such hindrances in respondents and their age group was noteworthy. Regarding technology, age groups agreed in most areas on its relevance, and rated insufficient system integration as the major obstacle. Only insufficient hardware was identified more frequently in group 1 compared to group 2, with a weak association with the nominal age.

Familiarity of the respondents with current trending topics regarding digitalization of the health care sector varied widely and seemed to decrease with recency of the discussion or initiative. Interestingly, age group 1 considered themselves to be less well informed across almost all topics. The association with the variable age was only relevant in the 3 topic areas of telematics infrastructure, the eHealth act, and the digital health care act.

More than half of the participants stated that they use mobile apps within their profession, with a weak tendency of an increasing adoption rate with decreasing age. Across all participants, most stated that the use of mobile apps was for information on pharmaceuticals, which was also weakly associated with age. Interestingly, the recommendation rate of mobile health apps to patients did not relate noticeably with the age (η=0.02), but was equally not very common. Only a share of 17.50% stated that they had recommended mobile health apps to patients. Insufficient services available was the main reason for not having done so yet for all participants, while insufficient data protection was a little more relevant for group 2 compared to group 1. The general belief of relevance of mobile apps for future health care provision was weakly associated with decreasing age.

The peer group “colleagues” as a source of information on mobile or digital health applications was slightly more important for younger respondents, while medical societies were the most relevant for all participants.

Respondents exhibited mainly positive expectations concerning the impact of digitalization on specific areas of health care. In particular, access to knowledge, medical research, and the treatment of rare diseases were associated with respondent optimism. Mixed feelings were expressed regarding the physician-patient relationship, administration, and attractiveness of the profession. The latter 3 categories, as well as medical quality, patient adherence, interdisciplinary collaboration, and early detection of diseases, showed a weakly increased optimism with the decreasing age of the respondents. The general attitudes toward upcoming changes through digitalization were split fairly evenly, with one-half having mainly positive feelings and the other having rather mixed feelings. The positive trend was once again weakly associated with age.

Regarding describing adjectives, being “critical” of digitalization was more associated with increased age, while being “open” was associated with decreased age. Indifference towards digitalization was hardly existent.

### Limitations

This study is subject to limitations due to participant selection and thus representativeness. With the first contact point being the digital channels of professional networks, a selection bias can be assumed [49]. Inherently, a digital contact point with a survey on digitalization itself might have led to a sample with a greater affinity for digitalization. On the other hand, the German Society for Tropical Medicine, Travel Medicine and Global Health e.V., for example, reaches 1060 of its 1085 members via email. We thus presumed that the undercoverage of respondents with no internet access could be ignored, since the self-organization of professional societies via digital channels can be assumed for the majority to be a prerequisite for professional participation. Another limitation might involve the initially mentioned self-administration of the health care sector in Germany. A presumed participant awareness of a potential interest of political stakeholders on such an investigation might lead to a tendency toward more extreme expression of opinion. However, we assumed this occurs in both directions.

Additionally, the partitioning of responses for the investigation of association was based on the age limit of the BJÄ and the definition of the German medical associations. To complement this presentation of results, a calculation of the eta coefficient incorporated the nominal value of age as an independent variable, where applicable.

### Conclusions

Physicians are emotionally involved in digitalizing health care provision, and they predominantly see opportunities as positive but also differentiated. The lower the involvement of second or third parties, such as patients or intersectoral service providers, was apparent, and the lower the GDPR sensitivity was assumed, and the higher was the apparent adoption rate of digital services. However, despite existing data security concerns, generic messaging apps were also found to be acceptable for professional communication from a quarter of the respondents, which supports the need for convenient and seamless solutions. Additionally, the need to personally perceive benefits through digitalization, like the saving of time, was expressed. Interestingly, this was more present with increasing age, which indicates an expectation of an automated and effortless transition. For younger generations, the handling of digital technology may be already inherent, and the conversion burden may thus not seem as onerous as that perceived by older colleagues. This theory might be supported by participants’ critical assessment of digital literacy or sovereignty as a field of development, which was increasingly perceived as a major hindrance for digitalization with increasing age. Query related to the current trending topics regarding digitalization of the sector confirmed the presence of an education gap. However, this was slightly more prevalent with decreasing age. Information and education on digitalization as a mean to support health care provision should thus not only be included in the course of study, but also in the continuing process of further and advanced training. Medical societies, statutory health insurance companies, and professional associations were mentioned as desired and trustworthy information providers. This also raises the question of determination and empowerment: when legislative initiatives are unknown, how does the profession want to participate in shaping digital health?

The role physicians see for themselves in the digitalization of health care provision was mainly described as “scrutinizing,” “active,” and “open.” This represents the ambivalence and inner conflict between observant expectation and active participation. The role of individual physicians as multipliers and stakeholders of digitalization within their scope of operation should be acknowledged, as well as the general willingness to participate in this process. On the other hand, the need for guidance and orientation through trustworthy organizations has a right to be instituted in the self-administered health care sector. The incorporation of physicians into the digitalization of their working environment is essential for a functional cocreation of future processes. However, digitalization is a multidisciplinary process [50], and despite the fact that digital affinity seems to increase in each successive generation, in a self-administered system, responsibility for this upcoming change cannot be attributed solely to physicians. A transformation of the system must be collaboratively implemented by all professional stakeholder groups, service providers and organizations, and political groups and sponsors.

## Appendix

Multimedia Appendix 1 Survey translation.

## Abbreviations

BJÄ: Bündnis Junge Ärzte (Alliance of Young Physicians)
DGV: Digitale-Versorgung-Gesetz (digital health care act)
DiGA: Digitale Gesundheitsanwendungen (digital health applications)
GDPR: General Data Protection Regulation
