# Supplementary material for: The Role of Physicians in Digitalizing Health Care Provision: Web-Based Survey Study
Source: JMIR Med Inform. 2021 Nov 11;9(11):e31527. doi: 10.2196/31527 (PMC8663562; doi:10.2196/31527)
Supplement: Multimedia Appendix 1 [file medinform_v9i11e31527_app1.pdf]

Multimedia Appendix

# Survey on the readiness for digitalization of physicians in the year 2020

Hello and welcome to the survey of the alliance of young physicians in cooperation with the Fraunhofer Institute for Software and Systems Engineering ISST with the focus on digitalization in the healthcare sector.

The year 2020 has it all!

Numerous laws and procedural changes have been implemented or are about to be implemented: In a very short time, things have been made up for that were long discussed but not implemented in the past years. To name a few examples: Telematics infrastructure, electronic health record, electronic prescription, electronic incapacity to work certificate, digital health applications (DiGA) and so on....

But how do all these changes affect physicians' work, how do these changes arrive in everyday life in practices, clinics and other places of healthcare delivery? How willing is the medical profession to use the numerous new digital applications and tools?

This is the core of our survey. The survey will take about 15 min to complete.

The survey is anonymous. For information on the processing of data please note the data protection notification. For reasons of better readability, the simultaneous use of the language forms male, female and diverse (m/f/d) is waived. All references to persons apply equally to all genders.

For any queries please reach out to umfrage(at)digitalisierte-medizin.de

We would like to take this opportunity to thank you for your participation.

Please click "Next" to start the survey.
There are 41 questions in this survey.

## Questions related to the person
### Demographics

Gender
☐ Male
☐ Female
☐ Diverse

Age

Professional level
☐ Medical student
☐ Physician in training
☐ Medical specialist <5 years experience
☐ Medical specialist >5 years experience
☐ Other:

Working environment
☐ Doctor’s office
☐ Clinic
☐ Public health service
☐ Not in patient care
☐ Other:

Occupation type
☐ Employed
☐ Self-employed
☐ Other:

Doctor’s office type
☐ Solo practice
☐ Joint practice
☐ Medical Service Center (MVZ)
☐ Other:

Doctor’s office leadership type
☐ Managed by a doctor
☐ Managed by a non-medical person

Sponsorship of the clinic
☐ University clinic
☐ Public
☐ Non-profit
☐ Private

Volume of employment
☐ Full-time
☐ Part-time
☐ Minor employment

Medical specialization
☐ General medicine
☐ Anesthesiology

☐ Anatomy
☐ Occupational medicine
☐ Ophthalmology
☐ Biochemistry
☐ Surgery - general and visceral surgery
☐ Surgery – vascular surgery
☐ Surgery - cardiac and thoracic surgery
☐ Surgery - pediatric surgery
☐ Surgery - oral and maxillofacial surgery
☐ Surgery - neurosurgery
☐ Surgery - plastic and aesthetic surgery
☐ Surgery – orthopedics and traumatology
☐ Geriatrics
☐ Gynecology
☐ Otorhinolaryngology and phoniatrics
☐ Dermatology and venerology
☐ Human genetics
☐ Hygiene and environmental medicine
☐ Internal medicine – general internal medicine
☐ Internal medicine - angiology
☐ Internal medicine - endocrinology and diabetology
☐ Internal medicine - gastroenterology
☐ Internal medicine - haematology and oncology
☐ Internal medicine - cardiology
☐ Internal medicine - nephrology
☐ Internal medicine - pneumology
☐ Internal medicine - rheumatology
☐ Paediatrics and adolescent medicine
☐ Child and adolescent psychiatry and psychotherapy
☐ Clinical pathology
☐ Microbiology, virology and infection epidemiology
☐ Neurology
☐ Nuclear medicine
☐ Public health
☐ Pathology
☐ Pharmacology
☐ Physical and rehabilitative medicine
☐ Physiology
☐ Psychiatry and psychotherapy
☐ Psychosomatic medicine and psychotherapy
☐ Radiology
☐ Radiotherapy
☐ Transfusion medicine
☐ Urology
☐ Other:

Attitude towards electronic devices and digital applications

| | Fully correct | Rather correct | Partly so, partly so | Rather not correct | Not correct at all |
|---|---|---|---|---|---|
| Electronic devices and digital applications facilitate my everyday life | ☐ | ☐ | ☐ | ☐ | ☐ |

| | | | | | |
|---|---|---|---|---|---|
| I find it easy to learn how to use an electronic device or an app | ☐ | ☐ | ☐ | ☐ | ☐ |
| I find digital applications and electronic devices reduce personal interactions between people | ☐ | ☐ | ☐ | ☐ | ☐ |
| I like to try out electronic devices and digital applications | ☐ | ☐ | ☐ | ☐ | ☐ |

**Status Quo**

In what way do you organize patient-related information in the following areas?

| | Completely paperbased | Predominantly paperbased | Equally paperbased and digital | Predominantly digital | Completely digital | Not relevant in my area |
|---|---|---|---|---|---|---|
| Patient admission | ☐ | ☐ | ☐ | ☐ | ☐ | ☐ |
| (Function) Diagnostic | ☐ | ☐ | ☐ | ☐ | ☐ | ☐ |
| OR | ☐ | ☐ | ☐ | ☐ | ☐ | ☐ |
| Intensive Care Unit | ☐ | ☐ | ☐ | ☐ | ☐ | ☐ |
| Care Unit | ☐ | ☐ | ☐ | ☐ | ☐ | ☐ |
| Patient discharge | ☐ | ☐ | ☐ | ☐ | ☐ | ☐ |

In what way do you exchange patient-related information?

| | Completely paperbased | Predominantly paperbased | Equally paperbased and digital | Predominantly digital | Completely digital | Not relevant in my area |
|---|---|---|---|---|---|---|
| Flow of information from other service providers to us | ☐ | ☐ | ☐ | ☐ | ☐ | ☐ |
| Flow of information from us to other service providers | ☐ | ☐ | ☐ | ☐ | ☐ | ☐ |

What mediums do you use for professional communication (both internally and externally)?

☐ Telephone
☐ Fax
☐ E-Mail
☐ Mail
☐ Medical platforms (e.g. referral portal, or other)
☐ General messenger-services / apps (e.g. WhatsApp, Telegram, ..)
☐ Specific medical messenger / apps (e.g. Siilo, Medione, Doctorsgate, ..)
☐ None of the mentioned mediums
☐ I don’t know
☐ Other:

In which areas do you use digital services to organize your everyday work?

☐ Professional training / e-Learning
☐ Duty / holiday planning
☐ Working time recording

☐ Personnel management
☐ None of the mentioned options
☐ I don’t know
☐ Other:

In which areas do you offer digital services to patients?
☐ Appointment scheduling
☐ Treatment management
☐ Digital information / anamnesis sheets
☐ Virtual consultation
☐ Electronic medication plan
☐ Electronic emergency dataset
☐ Access to personal data
☐ Remote querying of medical data (blood pressure, sugar,etc.)
☐ Digital health monitoring
☐ Mobile applications as therapy component
☐ None of the mentioned options
☐ I don’t know
☐ Other:

In which areas do you already see benefits through digitalization of your working environment and where do you see untapped potential for improvements through increased digitalization?
Please assess the current benefits in the columns on the left and the potential that is still available in the columns on the right.

| | No benefits noticeable | Benefits already noticeable | Untapped potential seen | No improvement expected |
|---|---|---|---|---|
| Data quality / readability of data | ☐ | ☐ | ☐ | ☐ |
| Availability of data across the entire process | ☐ | ☐ | ☐ | ☐ |
| Creation of data (e.g. through input masks or speech recognition) | ☐ | ☐ | ☐ | ☐ |
| Transparency for patients | ☐ | ☐ | ☐ | ☐ |
| Participation of the patient in the documentation | ☐ | ☐ | ☐ | ☐ |
| Flexibility of work organization | ☐ | ☐ | ☐ | ☐ |
| Reconciliation of private and working life | ☐ | ☐ | ☐ | ☐ |

Does it happen that patients bring in data recorded by consumer products (e.g. wearables, smartwatches, apps) for assessment?
☐ Never happened to me before
☐ Happens rarely
☐ Happens occasionally
☐ Happens regularly
☐ Other:

How do you deal with health-related digital data collected from patients themselves?
☐ I only check and consider acutely relevant, qualitative data
☐ I check and take into account all data that the patient makes available on his own initiative
☐ I reject all data from consumer-sources

☐ Other:

From your personal point of view and with regard to your colleagues of the same age group, what human aspects represent the greatest hindrances for the digitalization of healthcare?
☐ Lacking digital literacy/sovereignty
☐ Lacking willingness to change
☐ Lacking noticeable saving of time
☐ Fear of loss of importance
☐ Fear of surveillance
☐ I don’t see such hindrances in myself and my age group
☐ Other:

From your personal point of view and with regard to your colleagues of the same age group, what technical / organizational aspects represent the greatest hindrances for the digitalization of healthcare?
☐ Insufficient hardware equipment
☐ Insufficient software functionalities
☐ Insufficient system integration
☐ Too little budget for investment into digitalization
☐ Insecurity with regard to the legal frame of exchanging medically sensitive data
☐ Lacking cooperation by system providers
☐ None of the mentioned options
☐ Other:

Please rate your knowledge of the following topics related to digitalization on a scale from "never heard of" to "could explain it to a colleague who has no prior knowledge about it".

| | Never heard of | Heard before | Could I explain |
|---|---|---|---|
| Electronic health record | ☐ | ☐ | ☐ |
| Electronic patient record | ☐ | ☐ | ☐ |
| Telematics infrastructure | ☐ | ☐ | ☐ |
| E-health act | ☐ | ☐ | ☐ |
| Digital healthcare act | ☐ | ☐ | ☐ |
| Digital health applications (DiGa) | ☐ | ☐ | ☐ |

Does your workplace have secure Wi-Fi suitable for processing patient data?
☐ Yes
☐ No
☐ I don’t know

## Future

What changes do you expect from increasing digitalization in the following areas of healthcare?

| | Worsening | No change | Improving |
|---|---|---|---|
| Early detection of diseases | ☐ | ☐ | ☐ |
| Medical quality | ☐ | ☐ | ☐ |
| Access to knowledge | ☐ | ☐ | ☐ |
| Treatment of rare diseases | ☐ | ☐ | ☐ |
| Administration | ☐ | ☐ | ☐ |
| Patient adherence | ☐ | ☐ | ☐ |
| Doctor-patient-relationship | ☐ | ☐ | ☐ |
| Interdisciplinary collaboration | ☐ | ☐ | ☐ |
| Attractiveness of the profession | ☐ | ☐ | ☐ |
| Medical research | ☐ | ☐ | ☐ |

How do you face upcoming changes due to increasing digitalization
☐ Mainly positive

☐ With mixed feelings
☐ Mainly negative

How do you see your role in shaping the digitalization of healthcare?
☐ Active
☐ Contributing
☐ Reactive
☐ Waiting
☐ passive
☐ Critical
☐ Scrutinizing
☐ Indifferent
☐ Open
☐ Other:

## Mobile Health Applications

Do you use mobile applications in your everyday life as a doctor?
☐ Yes
☐ No

Which category can these apps be assigned to, and what are they called?
☐ Communication:
☐ Training:
☐ Information on pharmaceuticals:
☐ Diagnosis
☐ Other:

Do you trust in mobile health application?
☐ Yes
☐ No
☐ Depends

Have you ever used general communication apps (e.g. WhatsApp, Telegram, Threema, etc.) to exchange information about patients, e.g. to obtain a professional opinion or as an amendment to the shift handover?
☐ Yes
☐ No

Which communication channels have you already used for this purpose?
☐ SMS
☐ WhatsApp
☐ Threema
☐ Telegram
☐ Viber
☐ Other:

Have you ever recommended a mobile health app to a patient?
☐ Yes
☐ No

How many times have you recommended a medical app in the last quarter?

What is the name of the app(s) that you recommended?

Are there explicit reasons why you haven't recommended mobile health apps yet?
☐ Insufficient data protection
☐ In my opinion, apps are not suitable for my area of application
☐ No apps are sufficiently validated for my area of application
☐ I generally don't trust mobile healt apps
☐ Other:

Do you believe that medical apps will play a role in the treatment of your patients in the future?
☐ Yes
☐ No
☐ Other:

Why do you think medical apps will not play a role in the treatment of your patients in the future?

What kind of data sovereignty would you wish for in medical apps?
☐ Data sovereignty should lie with the patient
☐ Data sovereignty should lie with the physician if it contains treatment-relevant data
☐ Data sovereignty should lie with the physician AND the patient
☐ Data sovereignty can ly with the app provider

Which sources do you use to inform yourself about medical apps?
☐ Colleagues
☐ Internet
☐ Public bodies
☐ Medical societies
☐ App providers

Wherefrom would you like to get more information about digital health applications?
☐ Health fund physicians associations
☐ Medical chamber
☐ Employer
☐ Public bodies
☐ Medical societies
☐ Health insurance funds
☐ Professional societies and medical associations
☐ App providers
☐ I don’t know
☐ Other

If you have any further comments or wishes, please use the comment field:

Thank you for your participation.
You can now close the tab.
